# Marine Toxins Detection by Biosensors Based on Aptamers

**DOI:** 10.3390/toxins12010001

**Published:** 2019-12-18

**Authors:** Wei Ye, Taomei Liu, Weimin Zhang, Muzi Zhu, Zhaoming Liu, Yali Kong, Shan Liu

**Affiliations:** State Key Laboratory of Applied Microbiology Southern China, Guangdong Provincial Key Laboratory of Microbial Culture Collection and Application, Guangdong Open Laboratory of Applied Microbiology, Guangdong Institute of Microbiology, Guangdong Academy of Sciences, No. 100 Xianlie Middle Road, Yuexiu District, Guangzhou 510070, China; yewei@gdim.cn (W.Y.); liutm@gddcm.com (T.L.); zhumz@gdim.cn (M.Z.); Liuzm@gdim.cn (Z.L.); yaoyz@gdim.cn (Y.K.); 2014168125@ctgu.edu.cn (S.L.)

**Keywords:** marine toxins, aptamers, SELEX, biosensors, detection

## Abstract

Marine toxins cause great harm to human health through seafood, therefore, it is urgent to exploit new marine toxins detection methods with the merits of high sensitivity and specificity, low detection limit, convenience, and high efficiency. Aptasensors have emerged to replace classical detection methods for marine toxins detection. The rapid development of molecular biological approaches, sequencing technology, material science, electronics and chemical science boost the preparation and application of aptasensors. Taken together, the aptamer-based biosensors would be the best candidate for detection of the marine toxins with the merits of high sensitivity and specificity, convenience, time-saving, relatively low cost, extremely low detection limit, and high throughput, which have reduced the detection limit of marine toxins from nM to fM. This article reviews the detection of marine toxins by aptamer-based biosensors, as well as the selection approach for the systematic evolution of ligands by exponential enrichment (SELEX), the aptamer sequences. Moreover, the newest aptasensors and the future prospective are also discussed, which would provide thereotical basis for the future development of marine toxins detection by aptasensors.

## 1. Introduction

Seafood is very popular at home and abroad because of its delicious taste and rich nutrition. In recent years, with the improvement of people’s living standards and the rapid development of fishing and aquaculture industries, the consumption of seafood is also increasing. However, seafood poisoning incidents have become increasingly frequent, which has not only caused serious harm to the health and safety of people but also leads to huge economic losses to the seafood industry [1,2,3,4,5]. The marine toxin is one of the main factors causing seafood poisoning [6,7,8,9,10]. Marine toxins are mainly small molecular compounds with a wide variety and wide distribution. To ensure the safety of seafood, the detection of marine toxins in seafood has become increasingly important [11,12]. However, the existing traditional detection methods are difficult to take into account all the needs of sensitivity, simplicity, convenience, economy and so on, meanwhile, there is no sufficient rapid detection technology for on-site detection of marine toxins in seafood. Therefore, it is still urgently needed to develop rapid, sensitive, accurate and low-cost detection methods for marine toxins. In recent years, the rapid development of aptamer selection technology and biosensor fabrication technology offer a new solution for the detection of marine toxins with high efficiency and sensitivity [13,14,15,16,17].

Marine toxins are a class of small molecular compounds mainly produced by red tide algae. In recent years, with the aggravation of environmental pollution, human deaths due to accidental ingestion of toxic shellfish are also often reported [18,19,20,21]. At present, the main methods for the detection of marine toxins include mouse bioassay (MBA) [22], enzyme-linked immunosorbent assay (ELISA) [23,24,25], high-performance liquid chromatography (HPLC) and liquid chromatography–mass spectrometry (LC–MS/MS), etc. [26,27,28]. However, these traditional methods have some drawbacks including the requirement of a technician, poor repeatability, expensive equipment, and issues related to animal ethics [22]. As a new detection technology, the biosensor is a kind of analysis systems using cell molecules and other bio-materials as sensitive elements, combined with a secondary sensor to detect a variety of chemicals by cascade amplified signal [29]. Because of their advantages of simple operation, high speed, high sensitivity, miniaturization, and easy automation, biosensors have been widely used in different fields including cell physiology [30], drug screening [31,32], food safety detection [33], disease diagnosis [34], etc.

Aptamer, meaning “to fit”, is a kind of oligonucleotide which can bind to target molecules with high selectivity and affinity. On account of the existence of aptamers in nature and the possibility of being artificially isolated from pools of random nucleic acids, aptamers can bind to various kinds of compounds [35,36]. With the rapid development of systematic evolution of ligands by exponential enrichment (SELEX) technology, aptamers have been increasingly widely used in the detection of various kinds of chemical substances in different fields [37,38]. As a sensitive element, the aptamer not only reduces the detection cost but also shortens the detection time and enhances the detection sensitivity and specificity [39,40], and therefore biosensors based on aptamer have great potential in the detection of marine toxins.

## 2. The Toxicity of Representative Marine Toxins

### 2.1. The Representative Marine Toxins

**Figure 1:**
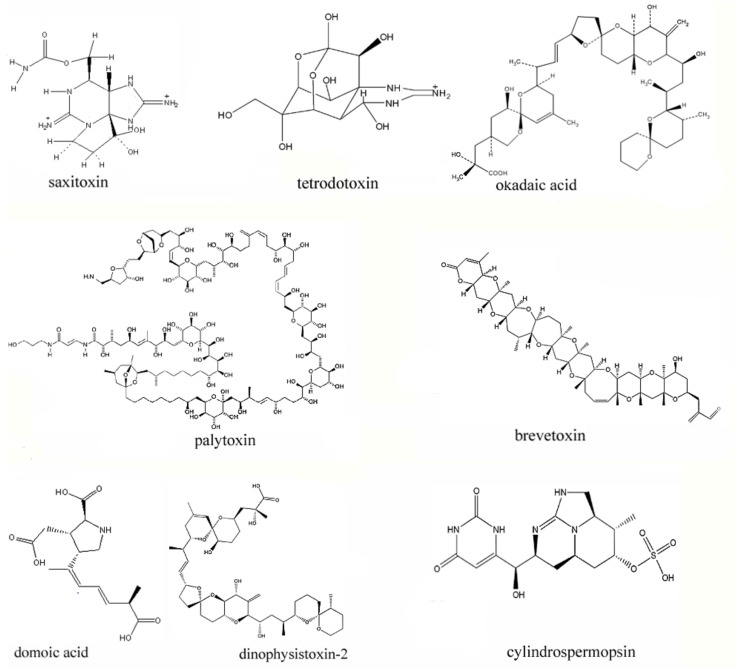
The structures of representative marine toxins.

Marine toxins can be enriched in shellfish tissues through the food chain, and the human ingestion of toxin-contaminated seafood can cause poisoning symptoms in different system organs [4,41]. The representative toxins include saxitoxin (STX), okadaic acid (OA), tetrodoxin (TTX), palytoxin (PTX), brevetoxin (BTX), domoic acid (DA) and dinophysistoxin (DTX) and cylindrospermopsin (CYN) etc. (Figure 1) [41]. 

### 2.2. The Toxicity of Representative Marine Toxins

Marine toxins cause great harm to human health through seafood intake [1,3,4,41]. Thus, it is of great significance to be aware of the toxicity of representative marine toxins, thus providing a thereotical guide for the alleviation of the hazard of marine toxins.

STX, a kind of marine toxin firstly isolated from bivalve molluscs of the clam genus, is a derivative of tetrahydropurine, the active sites are mainly in two agmatine groups and two hydroxyl groups [42,43]. STX is easily absorbed by the gastrointestinal tract, and whereas it cannot be destroyed by human digestive enzymes, STX is stable in high temperature and acidic solution which causes a great threat to human health [44]. However, the oxidation of STX under alkaline conditions can deprive it of its toxicity. The toxicity of STX is very strong, the mild toxic dose (LD_50_) to adults was 110 μg, the lethal dose was 540–1000 μg [45]. The toxic mechanism of STX is to inhibit nerve conduction by affecting sodium channels. STX can bind to toxin receptors on sodium channel proteins in neurons and myocytes, thus blocking sodium channels and leading to the disturbance of the neuromuscular conduction process and symptoms of random muscle relaxation paralysis in the human body [44].

OA is a kind of phycotoxin mainly produced by algaes including *Dinophysis* and *Prorocentrum*, which can be enriched in shellfish because of its feature of lipophilicity and heat-stablity [46], OA is one of the most widely distributed marine toxins that causes the highest incidence of events. LD_50_ (intravenous injection of mice) of OA was 166 μg/kg (body weight) [47]. The toxic mechanism of OA is complex. The results of epidemiological studies show that OA causes diarrhea by activating the intracellular cyclic adenosine monophosphate (cAMP) mediator system, that is, the increase of intracellular cAMP concentration leads to the activation of the protein kinase, phosphorylation, the secretion of a large amount of water, chlorine and carbonate, the inhibition of the normal absorption of sodium ions, and eventually leads to watery diarrhea. OA causes neurotoxicity even at the very low concentration of 0.5 nM, along with the symptom of degeneration of neuritis and cell body swelling, and then cell death. OA can inhibit the activity of PPl, PP2A catalytic subunit and make protein hyperphosphorylation in vivo, thereby causing a series of morphological and functional changes in cells, which in turn leads to diarrhea and promotes the development of carcinogenesis [48].

TTX, an amino perhydroquinazoline compound mainly found in puffer fish, salamander, *Xenopus*, etc., is one of the most toxic marine neurotoxins in nature. It acts rapidly on nerve endings and nerve centers after absorption, and can block sodium channels on nerve excitation membranes with high selectivity and affinity, thus causing nerve conduction blocking and resulting in nerve paralysis and death [49]. The acute oral toxicity of TTX was determined, and the LD_50_ of TTX towards mice by oral means was 10 μg/kg (body weight) [50].

PTX, a toxic polypeptide from *Anemonia sulcata*, can cause nerve and heart poisoning, and inhibit the activity of proteolytic enzymes. PTX causes cytotoxicity by activating cells to release potassium ions rapidly, leading to the symptom of hemolysis [51]. The lethal doses of PTX in guinea pigs, rabbits, dogs and monkeys by intravenous injection vary from 0.03 to 0.45 μg/kg. It is estimated that the lethal dose of PTX in humans is about 2.3–31.5 μg/kg [52].

DA is mainly produced by several members of the diatom genus *Pseudo-nitzschia* including *Nitzschia pseudodelicatissim*. DA can cause memory loss, vertigo, coma, and even death. The LD_50_ of DA towards mice is 10 mg/kg [53]. The toxic mechanism of DA is as follows: a. because the receptor binding efficiency of domoic acid to glutamic neurotransmitter is higher than that of glutamic acid, DA can also bind with the receptor of glutamic neurotransmitter. Thus nerve cells mistakenly assume that there is an excess concentration of glutamic acid, thereby resulting in the exclusion of all glutamic acid and the death of nerve cells. b. DA is an excitatory amino acid analog, which also competitively binds to two excitatory amino acid receptors and has a stronger affinity, thus opening sodium channels, resulting in abnormal influx of sodium ions and depolarization of the membrane and leading to the imbalance of sodium osmotic pressure. c. The channel opened by the binding of DA to the receptor is highly permeable to calcium ions, resulting in calcium influx and cell death [54].

BTX is mainly isolated from the dinoflagellate. This toxin can inhibit respiratory and myocardial function, resulting in spontaneous and repeated dose-dependent muscle contraction, leading to beam tremor, convulsion or jump, dose-related decrease in respiratory rate and bronchoconstriction of central and peripheral nerves. The LD_50_ of BTX-2 towards mice is 55.36 mg/kg [55], the toxicity of which is much weaker than that of shellfish-accumulated marine toxins including STX, PTX, TTX. BTX combines with the target site of sodium channel receptor and opening the sodium channel on the excited membrane, the permeability of cell membranes to sodium ion can be enhanced and the voltage-gated sodium channel can be activated. It produces strong cell depolarization and changes the conduction of neuromuscular excitation. Therefore, BTX has many toxic effects including embryotoxicity, developmental toxicity, immunotoxicity, genotoxicity and carcinogenesis [56].

Cylindrospermopsin (CYN) is a kind of alkaloid with good water solubility and heat stability, which is mainly produced by a large group of cyanobacteria. The LD_50_ of CYN towards mice by intravenous injection was 200 μg/kg. It exhibits a variety of toxic effects on human bodies by the exposure to cylindrospermopsin usually through the intake of water or food. The inhibition of CYP450 activity along with impairment of protein and glutathione synthesis resulted in the cell death of hepatocytes [57]. DA is a kind of marine toxin which can cause the sharp increase of intracellular free Ca, the change of cell energy metabolism and the overexpression of c-Fos protein in cultured rat hippocampal neurons *in vitro*, eventually leading to apoptosis or death of cultured hippocampal neurons [58,59]. It was reported that the LD_50_ of DTX-2 for mice was 338 μg/kg, the toxicity of which is about 60% as that of OA. As a derivative of OA, the toxicity mechanism of DTX-2 is inhibiting the protein phosphatase PP2A [60].

Given that the marine toxins are very harmful to human health, it is of great urgency to develop new detection methods of marine toxins in foodstuff or water with high sensitivity, specificity, convenience and relatively low cost, thus protecting humans from the toxicity of marine toxins.

### 2.3. The Development of Approaches for Marine Toxins Detection

As the development of molecular biology, chemistry and material sciences, great progress has been achieved in the detection methods of marine toxins, thus providing reliable criteria for the seafood safety.

#### 2.3.1. The Classical Detection Methods for Marine Toxins

Reliable and effective detection of marine toxins is an important prerequisite to avoid food poisoning, protect human health and reduce economic losses. The MBA method is time-consuming, laborious, and unable to distinguish the types of toxins, and the accuracy and repeatability are poor due to the differences of mouse breeds and individuals. What is more serious is that the method is also perplexed by experimental ethical and moral problems [22,61]. In vitro cytotoxicity analysis was achieved through microscopic observation of the morphological changes of experimental cells (such as neuroblastomas, mouse stem cells, human epidermocytoma cells, intestinal epithelial cells, fibroblasts, etc.), and 3-[4,5-dimethylthiazol-2-yl]-2,5 diphenyl tetrazolium bromide (MTT) color reaction [62]. The method of cytotoxicity analysis is simpler and more economical compared with MBA, but it is still time-consuming [62]. Moreover, the experimental results of different toxins are easily confused and are greatly influenced by subjective judgment. Instrumental analysis depending on chromatographic separation and ultraviolet (UV), fluorescence, mass spectrometry qualitative and quantitative techniques, includes gas chromatography (GC), high performance liquid chromatography (HPLC), liquid chromatography tandem mass spectrometry (LC–MS), capillary electrophoresis, and so on. The instrument analysis of marine toxins posses the advantages of relatively high sensitivity, low detection limit, good accuracy and repeatability, which has been used as an official detection method in many countries. However, the instrument analysis method has high requirements for sample pretreatment, high-purity standard products as a control, and also needs expensive instruments and equipment and professional technical personnel, which is not suitable for more and more on-site rapid testing [22,26,27].

Immunoassay is a mature field rapid method based on the specific recognition and binding of antigen and antibody. Among detection approaches, including enzyme-linked immunosorbent assay (ELISA), colloidal gold immunochromatographic strips have the advantages of simple operation, strong specificity and high sensitivity, does not need expensive instruments and equipment and professional operators, and is very suitable for on-site rapid detection and screening of actual samples, and some commercial ELISA kits have been put into practical application. However, the stability of antibodies is relatively low, the preparation of antibodies is tedious, time-consuming, and expensive [23,24]. Especially for small molecular substances such as marine toxins, the preparation of antibody is more difficult because of defects of their low immunogenicity, high toxicity, and cross-reaction [23,24,25].

#### 2.3.2. New Detection Methods Based on Marine Toxins Receptor

The biosensor is a kind of detection method using specific biomolecule recognition elements with specificity to interact with marine toxins and realizes the highly sensitive detection through specific signal transformation. Due to the miniaturization, portability and automation of related equipment, biosensors have a good application prospect in field detection and on-line monitoring [29]. Besides these conventional detection methods, different specific detection biosensors according to the characteristics of different marine toxins have also been developed.

For instance, by virtue of the ability of OA to inhibit protein phosphatase, enzyme activity inhibition approach to detect OA has been developed [63]. A receptor binding method to detect STX was developed taking advantage of the characteristics of STX specifically binding to sodium channel receptor [64]. Based on the characteristics of fluorescent substances produced by the reaction of TTX under alkaline conditions, a fluorescence method was developed for the detection of TTX [65]. The surface plasmon resonance (SPR)-based immunosensing method was employed to detect OA with a concentration of 12 μg per one kg shellfish mussel meat. The detection of OA in shellfish using luminescence of the lanthanide nanoparticles with the fluorophore-labelled antibody method showed detection limit of 0.25 μM. A sensing method based on luminescence resonance energy transfer combined with antigen-antibody recognition was also developed to detect toxins in shellfish, which shows advantages of high sensitivity and specificity. The method of surface enhaced micro-Raman scattering (SERS) with silver nanoparticles (AgNPs) was also developed to detect an extremely limited amount of lipophilic toxin [66]. This new method demonstrated merits of sensitivity, real-time detection, rapidity and relative low cost, however, the progress of SERS-AgNPs is hindered by the deficiency of database for marine toxins.

These new detection methods for marine toxins have their merits, but they cannot provide all the requirements of rapidity, simplicity, accuracy, sensitivity, and economy. Therefore, the development of rapid, sensitive, highly specific, accurate and low-cost marine toxin detection technology is still urgent. It is a research subject with important practical application value. In recent years, aptamer detection technology has developed rapidly. As a new recognition molecule, the aptamer can be used as a variety of detection methods instead of antibodies, and can also be employed as a recognition element of biosensors, thus opening a new avenue for the detection of marine toxins.

## 3. The Development of Biosensors Based on Aptamers

### 3.1. The Features of Aptamers

Aptamers refer to single-stranded oligodeoxynucleotides screened from synthetic single-stranded DNA (single strand DNA, ssDNA) or RNA libraries that can bind to target molecules with high affinity and specificity. The length of aptamer is generally 40–100 nt. Aptamers can adapt through intra-chain base complementary pairing and other intermolecular interactions. Conformational changes and three-dimensional folding to form relatively stable three-dimensional structures such as hairpin (hairpin), stem ring (stem-loop), convex ring (bulge loop), false knot (pseudoknot), G-quadruplet (G-quartet) and so on. These three-dimensional structures are the basis of the combination of aptamers and targets. When the target exists, the “lock key” is formed by matching the spatial conformation with the aptamer molecules. In this relationship, they form a stable combination through the stacking action, hydrogen bond action, ion bond action and electrostatic action of “pseudo base pair” [35,36,37].

Aptamer is a new type of recognition molecule, known as a “chemical antibody”, which has the following features [38,39,67]: (1) the binding affinity and selectivity of the aptamers to the targets are comparable to those of the antibody, and the dissociation constant (*K_d_*) can reach nM-pm range. (2) SELEX technology for screening suitable ligands is an in vitro screening technique that does not involve any animals or cells and is not contaminated by viruses and bacteria. Small molecular substances with toxicity and without immunogenicity can also be screened for high-affinity aptamers. (3) The selected aptamer sequences can be synthesized easily and duplicated by polymerase chain reaction (PCR) without relying on animals or cell experiments, in addition, the PCR procedure shows merits of short cycle, low cost, and small difference between different batches. (4) There are a wide range of targets for aptamers, ranging from small molecular substances such as amino acids, nucleotides, metal ions, toxins, organic dyes, antibiotics, cofactors, etc., to biological macromolecules such as enzymes, growth factors, antibodies, nucleic acids and proteins, and even complete viruses, bacteria and cells can be used as targets for aptamer screening. (5) The aptamer has good thermal and chemical stability and can be preserved for a long time and transported at room temperature. Even after denaturation, the aptamer molecule of oligodeoxynucleotides can be renatured under appropriate conditions. Therefore, the biosensor with aptamer as a recognition molecule can be used repeatedly, and its tolerance experimental conditions are more extensive. (6) The aptamer is convenient for functional group labeling or chemical modification, and the functional group can be introduced into the random library before, during or after screening, or the functional group can be labeled in the process of chemical synthesis to facilitate its detection and application. However, base coding into a random library may also increase the diversity and affinity of the sequence. (7) The molecular weight of aptamer is much smaller than that of antibody, has no immunogenicity, has strong tissue penetration, have good biocompatibility, can be used in drug delivery, molecular imaging and intracellular. (8) As oligodeoxynucleotides, the secondary structure of suitable ligands is easy to predict, and nuclease can be used. The tool enzyme can cut aptamer and modify it to improve its affinity and specificity, and it is also helpful for the development of flexible and diverse detection methods.

### 3.2. The Development of Biosensors

A biosensor is a new biological analysis and detection technology developed in recent decades, that uses functional organisms or biomacromolecules as primary recognition elements. The signals recognized by biomolecules are converted into electrical or chemical signals that can be measured through secondary transducers such as physical and chemical sensors. A biosensor has the advantages of good sensitivity and specificity, easy operation and miniaturization, so its application in marine toxin detection has attracted increasing attention [29,30,31,32,33,34].

The biosensors currently used for toxin detection can be divided into several categories, such as tissue sensors, cell sensors, enzyme sensors, optical sensors, electrochemical sensors, receptor sensors, immunosensors and DNA sensors. Since the emergence and development of SELEX technology, aptamers that can bind to target molecules with specificity, as a new sensitive element, has been widely used in the application of biosensors. In recent years, with the rapid progress of analytical technology, the bioanalysis model based on aptamers has also been developed. The biosensor (aptasensor) with suitable ligands has received more and more attention [39,40,68]. Aptamers show great advantages of repeatability, stability, simper preparation procedure, and low cost compared with antibodies, which can bind on the biosensors with high efficiency [38,67]. A surface plasmon resonance (SPR) biosensor is a new type of biosensor for the analysis of biomolecule interaction. Compared with the electrochemical method and fluorescence method, SPR has the advantages of non-labeling, high sensitivity and weight, good renaturation, low sample consumption, and wide detection range, and has a good application prospect in the fields of food safety, clinical diagnosis, drug targeting therapy [69]. A biolayer interferometry (BLI) biosensor is a biomembrane-dependent interferometric technique, this label-free and real-time monitoring technology could realize the determination of dynamic parameters of intermolecular interaction with the merits of being simple, fast, high throughput, of low cost and easily maintained [70]. It is also an effective alternative to ELISA, HPLC and SPR analytic methods. Biosensors based on surface enhaced micro-raman scattering (SERS) with silver nanoparticles (AgNPs) have also been exploited to detect the presence of OA and DTX in solutions and mussel tissues with high sensitivity and convenience.

### 3.3. The Calssification and Procedure of Aptamer-Based Biosensors

The analyte-specific aptamers show advantages of low lost, convenience, specificity, binding affinity, easy chemical modification and high sensitivity, thus allowing for the on-site rapid detection of toxins in food materials. The rapid development of biosensors offer great convenience for exploiting different kinds of aptamer-based biosensors to detect various analytes. Therefore, aptasensors have been widely used in the detection of toxins in foodstuff to ensure human health.

#### 3.3.1. The Classification of Aptasensors

According to the transduction signal mode, the aptamer-based biosensors can be devided into elctrochemical, optical, massed-based or calorimetric biosensors. The transduction signals of electrochemical biosensors are produced by a biochemical reaction between a DNA aptamer and target analyte, which yields or consumes ions or eletrons. And the materials of eletrodes are usually gold, compounds based on carbon or indium tin oxide. The conductometric electrochemical biosensors measure the alteration of electrical conductance of the aptamer-analyte reaction. Ion-charge effect biosensors detect ion concentrations employing ion-sensitive field-effect transistor, which consist of an ion-selective membrane allowing for the coupling of biorecognition element. The optical aptasensors determine the concentration of toxins by measuring the absorption, fluorescence, luminescence, internal reflection, light-scattering spectroscopy and SPR.

This optical aptasensors can be divided into label-free and label-based biosensors, the detection signal of the former is generated by the transducer, and the toxins lead to changes in the optical property. The label-based aptasensors take advantages of various optical probes and labels to generate luminescent, fluorescent or colorimetric signal resulting in the alteration of analytical wavelengths. Colorimetric sensors show merits of convenience, rapidity and on-site detection, which can be developed as portable detection biosensors for real-time inspection on toxins in different kinds of food.

#### 3.3.2. The Procedure for the Detection of Marine Toxins by Aptasensors

**Figure 2:**
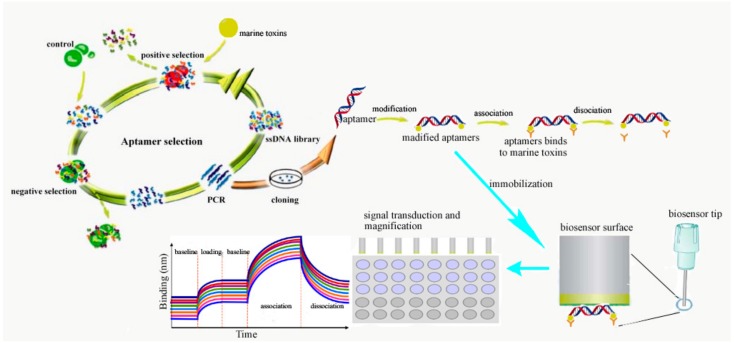
The detection of marine toxins by aptamer biosensors.

The detection of marine toxins by aptamer-based biosensors including the following procedure (Figure 2): (1) the screening of marine toxins aptamer by SELEX technology. The marine toxins are used as a target, enriched libraries are obtained after screening, and the optimized aptamer sequences are acquired by sequencing and affinity specificity analysis; competitive enzyme-linked ligand analysis (ELAA) based on nano-gold coloration was used to detect OA in the field. The aptamer OA27-1 screened by graphene oxide (GO)-SELEX was fixed on the microporous plate, the catalase labeled aptamer complementary chain was used to compete with the fixed OA aptamer. Different concentrations of H_2_O_2_ reduced chloroauric acid to produce gold nanoparticles with different aggregation states, showing different colors, in order to realize the colorimetric detection of OA [71]. (2) The establishment of an aptamer detection method, which includes: (1) aptamer detection technology based on fluorescence labeling, because when there is no target, the natural extension of the aptamer makes the fluorescent group and the quenched group far apart, resulting in the production of fluorescence detection signal. After adding the target, it can bind to the aptamer to form a stable hairpin structure, thus making the fluorescence group and quenching group close to each other, so as to realize the turn-off of the fluorescence detection mode. Because of the high cost and complex operation of labeling at both ends of the aptamer, researchers use carbon nanomaterials with the characteristics of adsorbed oligodeoxynucleotides and broad-spectrum fluorescence quenching as fluorescent receptors, which can avoid the labeling of quenched groups. It can also realize the simultaneous detection of multiple targets [65]. (2) Label-free fluorescence aptamer detection technique. A fluorescence method was established for the detection of cocaine by SYBR Green I and chain replacement amplification [72]. A cocaine aptamer was cut into two parts, a single-chain probe and a hairpin probe. In the existence of cocaine, it can combine with the two probes to form a triple complex, and the hairpin structure is opened and combined with the primers to initiate the chain replacement amplification reaction. The dsDNA formed can be combined with SYBRGreen I for fluorescence detection [72]. (3) The detection of marine toxins by an aptamer-based biosensor. Graphene oxide can immobilize different kinds of molecules because of its large specific surface area, thus could be utilized in the aptamer screening. The aptamer STX-41 screened by magnetic reduced graphene oxide (MRGO)-SELEX was connected with graphene quantum dots (GQDs). The fluorescence quenching system was established by the adsorption of MRGO [73]. STX binds to the aptamer to prepare the biosensor. The fluorescence detection of MRGO was realized by desorption and fluorescence recovery from the STX surface. DNase I can hydrolyze the aptamer that binds to STX, release STX, to recycle it, and combine with more aptamers to realize signal amplification. Under optimal conditions, the fluorescence aptamer analysis method can be used to detect 0.1–100 ng/mL with a STX, detection limit (LOD) of 0.1 ng/mL, which proves that this method can be utilized in the highly sensitive detection of STX [71,73].

#### 3.3.3. The Preparation and Optimization of Apatamer-Based Biosensors

The immobilization approaches for aptamers is of great importance for their stability and affinity, and therefore the immobilization of aptamers is vital for the preparation of aptamer-based biosensors. The physical or chemical absorption, inclusion, chemical cross-linking such as the 1-ethyl-3-(3′-dimethylaminopropyl)carbodiimide (EDC)/N-hydroxysuccinimide (NHS) method, micro-encapsulation and affinity binding have been utilized to immobilize aptamers. The principle of aptamer immobilization allows for the exposure of variable regions to ensure the selectivity, as well as the exposure of molecular regions that recognized by the analytes, in consequence, the process of aptamer immobilzation should not cause the great destruction of the target analyte-binding region of aptamers.

The marine toxin-coupling aptamers should be optimized to achieve the highest affinity and specificity. The optimization strategy of aptamers mainly includes truncation and mutation. Based on the secondary structure of aptamers predicted by mfold software, the non-essential nucleotides were truncated. At the same time, some bases were mutated with Q uadruplex-forming G-Rich sequences (QGRS) database in order to obtain a more stable spatial structure of aptamers, and BLI and SPR biosensors were used to measure the affinity and specificity of aptamers [74,75].

Meanwhile, the detection process by aptamer based-biosensors should also be refined to obtain the highest signal noise ratio. For instance, the biotin modified aptamer was immobilized on the sSA chip and coupled with different concentrations of BTX. The standard curve was established by detecting the change of signal by BLI. According to the shift of the response value of the aptamer sensor under different detection conditions, the detection time, the composition of the buffer, the pH value and the concentration of the aptamer were optimized in order to acquire the best detection system. On this basis, the fitting curve between BTX concentration and response value was obtained, and the linear range of detection (LRD) and LOD of the sensor were further analyzed [76,77]. Different concentrations of BTX standards were added to sea water and shellfish and other food samples, and the aptamer-based biosensor was used to detect the marine toxins in different samples.

Moreover, the safety of sea food is drawing increasing attention currently because of the great toxicity of marine toxins, thereby lower and lower concentrations of marine toxins in sea food are in demand to ensure human health. The rapid development of nanomaterials including single-walled carbon nanotubes, titanium dioxide nanotubes, graphene oxide nanosheets, graphene hydrogels, lanthanide nanoparticles, quantum dots and gold nanoparticles have also been utilized to enhance the sensitivity and LOD of aptasensors.

## 4. The Detection of Marine Toxins by Aptamer-Based Biosensors

### 4.1. The Detection of Representative Marine Toxins by Aptasensors

The representative marine toxins cause great threats to human health by the intake of seafood [2,4]. Hence, it is urgent to develop new detection methods. Biosensors based on aptamers have been widely used in marine toxins detection by virtue of the merits including high sensitivity, stability and specificity.

The STX-aptamer complex was resuspened in solution, the other ssDNAs were absorbed by magnetic reduced graphene oxide (MRGO), then the aptamer STX-41 for saxitoxin was screened and multiple-cycle amplified [78]. The fluorescence quenching system was established by graphene quantum dots (GQDs) connection and adsorption of MRGO using aptamers for saxitoxin. The MRGO was added to the mixture of carboxyfluorescein (FAM)-labeled aptamers and saxitoxin to quench the fluorescence of uncombined labeled saxitoxin. Then the concentration of saxitoxin can be determined by the fluorescence intensity. Under the optimal conditions, the fluorescence aptamer analysis method can detect STX with LRD of 0.1–100 ng/mL and LOD of 0.1 ng/mL using aptamer STX-41 [78]. This method showed the advantages of relatively high sensitivity and specificity. However, the LOD still can be improved by the optimat the instrument for the fluorescence detection is still needed, which is not so adequate for on-site rapid detection of marine toxins.

ssDNA aptamers that show high specificity and affinity for OA were obtained after 18 rounds of target and negative target selection. The candidate aptamer with the highest affinity (*K_d_* = 77nM) was chosen for circular dichroism analysis. A conformational change in the aptamer was observed upon binding of OA. A label-free electrochemical impedimetric biosensor was developed using this aptamer and achieved a LOD of 70 pg/mL. Meanwhile, no cross-binding activity toward structurally similar toxins was observed, including dinophysis toxins-1 and -2 and microcystin-LR [79]. Moreover, a ssDNA aptamer that specifically binds to OA with high affinity was obtained employing SELEX technology by the assistance of graphene oxide (GO), and a novel competitive ELAA approach was developed using selected aptamer. This detection method for OA showed a low LOD of 0.01 ng/mL, wide linear range (0.025 to 10  ng/mL), and high recovery rate (92.86–103.34%) in OA-contained clam [71]. Overall, graphene oxide has been used to assist the selection of optimal aptamer with high affinity for OA, electrochemical impedimetric biosensor and ELAA were employed to detect OA with low LOD to pg and high specificity, which can facilitate the sensitive detection of OA, thus alleviating the hazard of OA towards human health.

A 78-mer ss DNA library was synthesized in vitro by Shao et al. A TTX-specific monoclonal DNA aptamer A3 was prepared using SELEX combined with mutagenic PCR by screening, enrichment, cloning and sequencing. The secondary structure of the DNA aptamer A3 mainly contained a stem ring structure, and the affinity for TTX was 1.254 nM. The optimized results indicated that the optimal buffer pH was 7.5 and the best fluorochrome-binding time was 10 min. As a result, a DNA aptamer fluorochrome method for rapidly screening and detecting TTX was developed with a LOD of 1 μM [80]. The aptamer for TTX shows high affinity, however, no label-free biosensor for TTX detection has been developed until now, leading to the LOD for TTX being less than ideal. The devleopment of label-free aptasensor including electrochemical impedimetric sensor, SPR sensor, graphene quantum dots is impulsive to improve the detection sensitivity for TTX.

A biosensor was developed by BLI coupled with competitive binding assay through an enzyme-linked aptamer to detect palytoxin with advantages of high sensitivity, speed and on-site detection. Aptamers labeled with horseradish peroxidase were used as to competitively bind to palytoxin. The recipitated polymeric product on the surface of biosensor formed by PTX-horseradish peroxidase-aptamer complex caused a remarkable shift in the biosensor layer’s optical thickness, which significantly changed the interference pattern and led to a response profile on the surface of BLI biosensor. The biosensor displayed a wide linear range of 0.2–0.7 ng/mL, very low LOD of 40 fg/mL for PTX. In addition, the biosensor was then utilized to the detect PTX in spiked extracts with the merits of high selectivity, repeatability, and stability. This aptamer-based biosensor would offer a sensitive and selective detection method for PTX [81]. The aptasensor using BLI coupled with labeled aptamers for PTX showed a very low detection limit, we believe lower LOD can be obtained with the development of new biosensors and aptamer-screening approaches.

It was reported that a graphene functionalized sensing-based biosensor combined with a quartz crystal microbalance immunosensor was used t to detect BTX [82]. A dendrimer decorated with gold nanoparticles was used to fabricate electrochemical immunosensors to detect BTXs [83]. Moreover, Tang et al. have developed guanine-functionalized graphene nanoribbons [84]. However, there are demerits in these immunosensors including the instability, high cost and tedious production procedure of antibody preparation, which would impede the widespread application of these immunosensing approaches in detecting BTXs. The disadvantages of conventional BTX-detection methods encouraged researchers to excavate new detection methods for BTX with convenience and high sensitivity. Using in vitro selection, an aptamer for BTX-2 with a high binding affinity of 42 nM was selected from a large pool of random sequences. The incubation time, pH and metal ions concentrations for the aptamer-toxin binding affinities were optimized. A label-free competitive impedimetric biosensor used aptamer BT10 to detect BTX-2 with a very low LOD of 0.106 fg/mL. The aptamer-sensor was applied in the detection of BTX-2 in spiked shellfish extract and displayed a very high recovery [85]. However, the SELEX approaches should be optimized to obtain aptamers with a higher specificity and binding affinity for BTX-2, and a lower LOD to 10 pg/mL level for BTX-2 detection could be realized by the utilization of sensors including graphene quantum dots, SPR or BLI, etc.

A ssDNA aptamer with high affinity of 88.78 nM and high selectivity for CYN was selected using SELEX technology. Circular dichroism measurements results indicated that a conformational change was detected when this aptamer binding to CYN. This phenomena was utilized to develop a label-free impedimetric biosensor, the LOD of which was 100 pM, and the linear range of which was 100 pM–80 nM. Almost no responses was detected for the coexistence of cyanobacterial toxins including microcystin-LR and anatoxin-a, demonstrating the high specificity of aptasensor [86]. However, the aptamer with higher affinity can be acquired by the assistance of graphene oxide, thus realizing a lower LOD of CYN detection by an aptasensor.

There are only very few reports on the aptamer screening for DTX marine toxins. The SELEX screening of toxin-specific nucleic acid aptamers was carried out by using magnetic beads or graphene oxide (GO) to immobilize toxins and other fixed nucleic acid libraries. Aptamers with strong selectivity and high binding affinity for BTX, DTX, nodularin (NDR) and microcystin (MC) were obtained. Through molecular calculation and structure simulation, the structural basis of toxin binding to aptamers was analyzed, and the optimization of aptamer sequences and structural strategies was established. BLI and SPR were introduced to construct a novel sensitive, specific and fast approach for the detection of marine toxins based on aptamer biosensors. It has been preliminarily confirmed in cells and mice that some adaptations can block the hemolysis and toxicity of specific toxins [87].

Based on the characteristics of magnetic separation and adsorption of single-strand oligodeoxynucleotides by MRGO, after several rounds of screening by SELEX, a number of aptamer candidate sequences for DA were obtained, the affinity specificity of which was analyzed by fluorescence method. The aptamer DA-06 with high affinity specificity to DA (*K_d_* = 62.07 ± 19.97 nM) was obtained [78]. However, this is no report on the aptamer based biosensor for DA unitil now.

LC-MS was also adopted to detect the existence of OA, DTX-1, PTX-2 and yessotoxin in shellfish. The results show that the linear range of PTX-2 was 1.0–100.0 μ g/L, the quantitative limit was 0.5 μg/kg, and the relative standard deviation (RSD) was 3.16–9.29% [88]. An approach using BLI coupled with ELAA biosensor for the detection of PTX displayed wide linear range (0.2–0.7 ng/mL), a very low LOD (0.04 pg/mL), implying the great advantages of the detection of marine toxins by aptamer-based biosensors.

Microcystin is a kind of hepatotoxin mainly produced by cyanobacteria. cDNAs were immobilized on AuNPs, and the polymeric AuNP network complex that was built containing the aptamer and cDNA of microrocystin LR containing a leucine substituent (MC-LR) was detached through the addition of the MC-LR toxin. The capture rate of the immobilization of the aptamer on the AuNPs increased, along with an improved signal-to-noise ratio, which was owing to the increased volume and decreased surface charge. The selected aptamers exhibited binding affinity from 0.1 nM to 20 μM for MC-LR. Aptamers for three kinds of different microcystin analogs including microrocystin LR containing a leucine substituent were selected by SELEX and immobilized on biosensor. However, SPR binding results suggested the higher binding affinity between the target aptamer and microcystin YR, which is an analog containing a tyrosine substituent for microcystin. Significant binding was also observed between the aptamer and microcystin RR, which was an analog containing an arginine substitutent for microcystin. The binding affinity of the aptamer and microcystin RR was low, the Ka of which was approximately 10^3^ M^−1^ [89,90], which did not demonstrate the high affinity and specificity properties of aptamers. However, this selected aptamer shows the potential of being used as a molecular recognition element (MRE) in a label-free biosensor. A detection strategy for MC-LR using the nanopore approach is proposed by He et al. based on the interaction between aptamer and host-guest utilizing a gold nanoparticle (AuNP) probe. The aptamer of MC-LR and its complementary DNA (cDNA) are respectively immobilized on AuNPs with distinct sizes, and the constructed polymeric AuNP network via the hybridization of the aptamer and cDNA was disintegrated upon the addition of MC-LR. [91]. Aptamers for microcystin-LR, microcystin-YR and microcystin-LA were obtained with *K_D_* of approximately 50 nM. The electrochemical aptasensors based on selected aptamers could detect microcystin and its congeners with very low LOD of 10 pM [92,93], demonstrating the great advantages of the detection of MC-LR by aptasensor. The toxicity, toxic mechanism, the lowest LOD, linear range of detection (LRD), aptamer sequence, immobilization method and biosensor type of representative marine toxins are summarized as follows (Table 1):

### 4.2. Recent Advances in Detecting Toxins by Aptasensors

Various efforts have been made to improve the detection limit and specificity of aptamer-based biosensors. Only a few reports are available on the progress in aptasensors for marine toxins detection, thus the latest progress for the improvement in aptasensors for detecting different toxins are summarized, providing new insights into the improvement of marine toxins detection by various aptasensors.

A lab-on-a-disc (LOAD) system can control and manipulate small quantities of liquids contained accurately within a lab-on-a-chip (LOC) platform. The LOAD-based platform combined with immunofluorescence by centrifugally driven microfluidic liquid handling was used to detect MC-LR, DA and STX in freshwater in situ within 30 min. The LODs for MC-LR, STX and DA detection by the LOAD-based platform were 7.2 ng/mL, 50 ng/mL and 20 ng/mL [94], respectively. This approach shows advantages of relatively low cost, in site detection, no requirements for expensive instruments, thus offering a reliable method for the in-situ toxin detection high-efficiency. However, the LOD could be enhanced by the optimization of aptamers for these toxins. The stability and the high cost of the antibody for MC-LR is still a great challenge for wider application.

To enhance the detection sensitivity and selectivity, aptamer-conjugated quantum dots (QDs) absorbed on Au nanoparticles with a quenching effect on QDs was utilized to detect aflatoxins in peanut and rice, the detection limit of this nano-aptasensor was 3.4 nM, and the linear range was 10–400 nM, the results indicated that this aptamer based nanobiosensor showed a bright prospect in the detection of different toxins in complex samples [95]. Total internal reflection ellipsometry based on SPR in nano-structured gold films was developed to detect aflatoxin B1 and M1 with low LOD of 0.01 ng/mL, and LRD of 0.01–100 ng/mL. The affinity constant of afflatoxins to their respective aptamers was 10^−8^–10^−7^ mol, which is similar to their monoclonal antibody [94]. An aptamer-based SPR biosensor was exploited to detect aflatoxin B1 and aflatoxin B2 simultaneously, the streptavidin was immobilized on the CM5 chip and the biotin-labeled aptamer was captured to detect aflatoxin B1 and B2. This SPR biosensor showed LRD from 1.5 to 50 ng/mL and LOD of 0.19 ng/mL [96]. An thiol-modified aptamer with specific binding affinity for MC-LR was developed, then immobilized on a gold surface covalently, this aptamer-based biosensor displayed LRD of 1–50 μg/mL and could distinguish MC-LR from other congeners, which has great background for the detection of different cyanotoxins by developing various aptamers with high binding affinity and selectivity [97]. Ochration A (OTA) is a common mytoxin in foodstuffs. A rapid and sensitive fluorescence-based aptamer biosensor was exploited by Wu et al. The OTA could form a structure of G-quadruplex with thioflavin T dye and led to increased fluorescence, and only faint fluorescence was observed after the formation of an OTA-OTA aptamer-G-quadruplex structure by joining OTA. The aptamer based biosenor showed low LOD (0.4 ng/mL) and good LRD of 1.2–200 ng/mL, which provide promising detecting approach for mytoxins in different foodstuff [98]. Aptamer antibody pair-based lateral flow assays using aptamer-coated AuNPs biosensor were employed to detect cholera toxin with low LOD of 0.6 ng/mL, thus offering a promising tool for the phathogen toxins [99].

**Figure 3:**
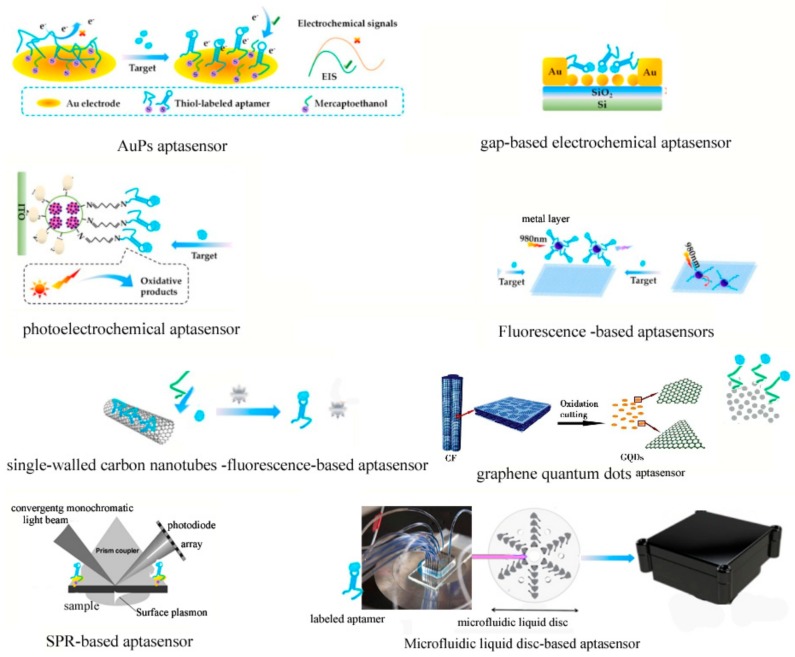
The scheme of newest developed aptamer-based biosensors.

DNA aptamers have wide application in biosensors because of the stability, specificity and high affinity for chemicals. However, DNA aptamers also have drawbacks of lacking wide array of computational tools. An approach predicting from sequence to 3D structures of ssDNA for aptamer application was firstly reported by Jeddi et al. The approach integrates 3D ssRNA models to ssDNA 3D structures and refines the resulting ssDNA 3D structure, which could faithfully predict the representative nucleic acid database, thus opening a new avenue for the integration of DNA computational analysis with aptamer-based biosensors designing [100]. Many efforts have been made to enhance the sensitivity and selectivity of biosensors based on aptamers [101], however, little attention have been focused on the improvement of the response rate of biosensor. The sensible engineering of a quickly responsive and highly sensitive aptamer-cDNA probe was realized by utilizing bivalent interaction of two short monovalent cDNA sequences, then the probe was hybridized simutaneously to two electrode-imobilized aptamer-based probes combined with eletrochemical aptamers, which could enhance the response rate from 30 min to 4 min [102,103]. The latest developed aptasensors are summarized in Figure 3.

## 5. Conclusions and Future Perspectives

Marine toxins cause great harm to human health through the intake of seafood, thereby prompting researchers to exploit new marine toxins detection methods with high sensitivity and selectivity, quickness, low LOD, convenience and high efficiency. The rigorous requirements of equipment, standard and professional engineers for LC-MS detection approach limited its wide application in marine toxins detection. The laborious procedure and high cost of marine toxin detection methods by antibody restrict their wide use. The MBA methods should take into account ethical and moral issues. With the rapid development of molecular biological approaches and the sequencing technology, the screening of aptamers for marine toxins by SELEX is more and more economical, convenient and time-saving. Moreover, the fast development of material sciences and chemical processes boost the preparation and application of biosensors. Taken together, the aptamer-based biosensors would be the best candidate for marine toxins detection with the merits of high sensitivity, convenience, being time-saving, of relative low cost, having an extremely low detection limit, and high throughput.

As the progress in the SELEX technology, aptamers are easier to screen with lower cost and more convenience. RNA aptamers have limited application because of their lower stability and higher cost. Single-stranded DNA aptamers possess several advantages including stability, reusability, sensitivity, easy to suffer chemical modification and lower production cost over antibodies. However, there are also limitations for ssDNA aptamers, and the binding affinities of aptamers are closely related with their three-dimensional structures, which are affected by various factors including the ionic condition, pH, and temperature of the binding condition [103]. The cross-binding activities to other similar molecules in natural environments remain a great challenge for marine toxins detection by aptamers, which would hinder the application of aptamers in marine toxin detection in shellfish food or algaes samples. Therefore, it is crucial to enhance the affinity and specificity of identified aptamers to desired marine toxins by careful design of the SELEX method and the modification of bases in PCR amplification or chemical modification to resist the attack of nucleases in different biological samples, thus promoting the development of aptamer-based sensors and their application in increasingly diverse fields. The coating procedure of aptamers for marine toxins on different materials including gold nanoparticles and graphene quantum dots should also be optimized, which would reduce the costs of aptamers, ensure the exposure of binding-regions and variable regions, retain and binding affinities of aptamers thereby enhancing the detection sensitivity of aptamer-based biosensors. The optimal labels for aptamers and transduction materials for aptasensors should also be elaborately refined to achieve the highest signal and lowest signal noise, thus realizing the lowest LOD for marine toxins by aptasensors. Considering the demand for spot rapid diagnosis of different complex samples, the prospect of biosensors based on portable ssDNA aptamers would be bright in a variety of settings, such as food safety, environmental pollution monitoring, and human health. The prominent merits of ssDNA aptamer biosensors would prompt researchers to focus on the investigation on the optimization of aptamers screening and the fabrication of new aptasensors constantly.

Although aptasensors have been widely used in different fields [102,103,104,105,106,107,108], great efforts still need to be made in the following respects: (1) the optimization of aptamer selection process. Although some aptamers for marine toxins have been selected, it is still not sufficient for a large amount of marine toxins with categories of more than 1000. GO-assisted SELEX can accelerate the SELEX process because of convenience and high efficiency. Moreover, the capillary electrophoresis-SELEX and microfluidic SELEX combined with microfluidic chip technology would promote the screening of aptamers for various kinds of marine toxins; (2) despite the stability and high affinity of aptamers, there is still no adequate tools to optimize the aptamers according to their structures. With the rapid development of bioinformatics, more tools would be exploited to refine the aptamers for marine toxins to achieve higher specificity and affinity. (3) Aptamer-based biosensors have wide applications in toxins’ detection, however, there still remains much scope for the enhancement of the sensitivity, selectivity and response rate of aptasensor. Since the emergence of biosensors including QDs, AuPs, GO, photoelectrochemical biosensor, gap-based aptasensor [109] fluorescence-based aptasensor and single-walled carbon nanotubes [110], the marine toxins detection by aptasensors made considerable headway. With the vigorous development of bioinformics, molecular biology, chemistry, and material science, the aptamer-based biosensors would play a more and more important role in the detection of marine toxins. The aptasensors based on optical signal would be increasingly convenient and high-efficient by the utilization of mobile phone applications. Given the intense alteration of and biodiversity in marine life, an increasing number of marine toxins are emerging, which calls for detection approaches with high throughput, high sensitivity and convenience to fabricate and modification. Overall, by virtue of these requirements, aptasensors will have a more and more promising future in the marine toxins detection by the combination of aptamer modification and newly developed biosensors.

## Figures and Tables

**Table 1:** The classification, toxicity, toxic mechanism and aptamer-based detection of marine toxins.

Marine Toxins	Toxicity (LD or LD_50_) Towards Mice	Toxic Mechanism	Aptasensor Type	LOD	LRD	Aptamer Sequences	The Immobilization Method	Reference
STX	LD_50_ = 10 μg/kg	binds to sodium channel proteins	graphene quantum dots	0.1 ng/μL	0.1–100 ng/μL	CTTTTTACAAAATTCTCTTTTTACCTATATTATGAACAGA	Physical absorption of MRGO	[42,43,44,45,73,77,78]
TTX	LD_50_ = 10 μg/kg	blocks nerve conduction	aptamer fluorochrome (EvaGreen)	1 μM		TCAAATTTTCGTCTACTCAATCTTTCTGTCTTATC	___	[49,50,78,80]
OA	LD_50_ = 166 μg/kg	activating cAMP mediator system, inhibiting PP1A, PP2A	Fluorescence combined with rolling cycle amplification	10 pg/mL	0.01–100 ng/mL	GGTCACCAACAACAGGGAGCGCTACGCGAAGGGTCAATGTGACGTCATGCGGATGTGTGG	The binding of biotin-labeled aptamer to streptavidin-catalase complex	[46,47,71,78,79]
PTX	LD = 2.3-31.5 μg/kg	activating cells to release potassium ions rapidly	Biolayer Interferometry (AR2G biosensor)	0.04 pg/mL	200–700 pg/mL	ACCGACCGTGCTGGACTCAGGAGGTGGTGGGGACTTTGCTTGTACTGGGCGCCCGGTTGAAACTATGAGCGAGCCTGGCG	EDC/NHS method	[51,52,81]
BTX	LD_50_ = 55.36 mg/kg	open and activate sodium channel	label-free impedimetric biosensor (electrochemical biosensor)	106 pg/mL		GGCCACCAAACCACACCGTCGCAACCGCGAGAACCGAAGTAGTGATCATGTCCCTGCGTG	The BTX was immobilized on cysteamine-modified gold electrodes	[55,56,85]
DA	LD_50_ = 10 mg/kg	bind with the receptor of glutamic neurotransmitter	___	___		___		[53,54,58,59,78]
DTX-2	LD_50_ = 338 μg/kg	inhibiting PP2A	___	___	___	___		[60,88]
CYN	LD_50_ = 200 μg/kg	Inhibit the synthesis of protein, glutathione and P450 activity	Label free impedimetric biosensor (Graphene-Based biosensor)	1.9 pM		GGCATCAGGCAACAACCGATGGTCCGGCCACCCTAACAACCAGCCCACCCACCACCCCGCCG	utilizs an unlabeled aptamer noncovalently assembled on a graphene electrode	[85,86,87]

“_” means no aptamer sequences were reported.

## Abbreviations

AgPs	silver nanoparticles
AuPs	gold nanoparticles
BLI	biolayer Interferometry
BTX	brevetoxin
cAMP	cyclic adenosine monophosphate
CYN	cylindrospermopsin
DA	domoic acid
DTX	dinophysistoxin
EDC	1-Ethyl-3-(3′-dimethylaminopropyl)carbodiimide
ELAA	enzyme-linked ligand analysis
ELISA	enzyme-linked immunosorbent assay
FAM	carboxyfluorescein
GC	gas chromatography
GO	graphene oxide
HPLC	high-performance liquid chromatography
LC-MS/MS	liquid chromatography-mass spectrometry
LD_50_	mild toxic dose
LOAD	lab-on-a-disc
LOC	lab-on-a-chip
LOD	limit of detection
LRD	linear range of detection
MBA	mouse biosassay
MC-LR	microrocystin LR containing a leucine substituent
MRE	molecular recognition element
MRGO	magnetic reduced graphene oxide
MTT	3-[4,5-dimethylthiazol-2-yl]-2,5 diphenyl tetrazolium bromide
NDR	nodularin
NHS	N-Hydroxysuccinimide
OA	okadaic acid
OTA	Ochration A
PTX	palytoxin
QDs	quantum dots
QGRS	q uadruplex-forming G-Rich Sequences
RSD	relative standard deviation
SELEX	systematic evolution of ligands by exponential enrichment
SERS	surface enhaced micro-Raman scattering
SPR	surface plasmon resonance
STX	saxitoxin
UV	ultraviolet
